# Lactating Vulvar Adenoma Associated With Fibroadenoma

**DOI:** 10.1177/10668969251314125

**Published:** 2025-02-17

**Authors:** Samuel Robichaud, Danh Tran-Thanh, Zhi Ping Zhang, France Durocher, Kurosh Rahimi

**Affiliations:** 1 Department of Pathology, 5622 Université de Montréal, Montreal, Quebec, Canada; 2 Departement of Pathology, 59429 Cité-de-la-Santé Hospital, Laval, Quebec, Canada; 3 Les Gynécologues de Laval, Laval, Quebec, Canada

**Keywords:** vulvar fibroadenoma, ectopic breast tissue

## Abstract

Ectopic breast is found to occur most commonly in the axilla, but also less commonly on the vulva and on sites outside the “milk line” like the face, neck and chest. Vulvar adenofibroma developing on ectopic breast tissue in the vulva is exceptionally rare. More precisely, vulvar adenofibroma associated with adenoma with lactating changes has only been documented three times to our knowledge. We herein report a vulvar mass specimen consistent with vulvar lactating adenoma associated with vulvar fibroadenoma occurring in a 36-year-old woman in the postpartum period.

## Introduction

Ectopic breast tissue can occur along the embryonic milk lines found between the axilla and the medial side of the groin.^1^ It has an incidence of around 1% to 3% in women and can also occur, albeit less frequently, in men.^2^ These lesions are subject to hormonal influences and can develop into lesions also found in the breast, spanning from benign entities such as fibroadenomas and papillomas to carcinomas.^3,4^

## Case Report

A 36-year-old woman with history of endometrial polyps presented with a vulvar mass during the postpartum period. Physical examination showed a painful 2 × 1 cm skin colored mass on the posterior fourchette of the vulva. The patient later underwent surgical excision of the lesion. The grossing specimen revealed a 2.5-cm mass containing whitish liquid and numerous vegetations.

On histology, the lesion consisted of mammary-like glands with secretory and cystic changes. The cystic spaces were lined by cuboidal cells with secretory changes. Focally, a biphasic fibroepithelial proliferation was noted consisting of a simple cuboidal epithelium surrounded by a loose stromal component. No cytonuclear atypia, tumor necrosis or significant mitoses were noted in any of the components.

**Figure 1.**
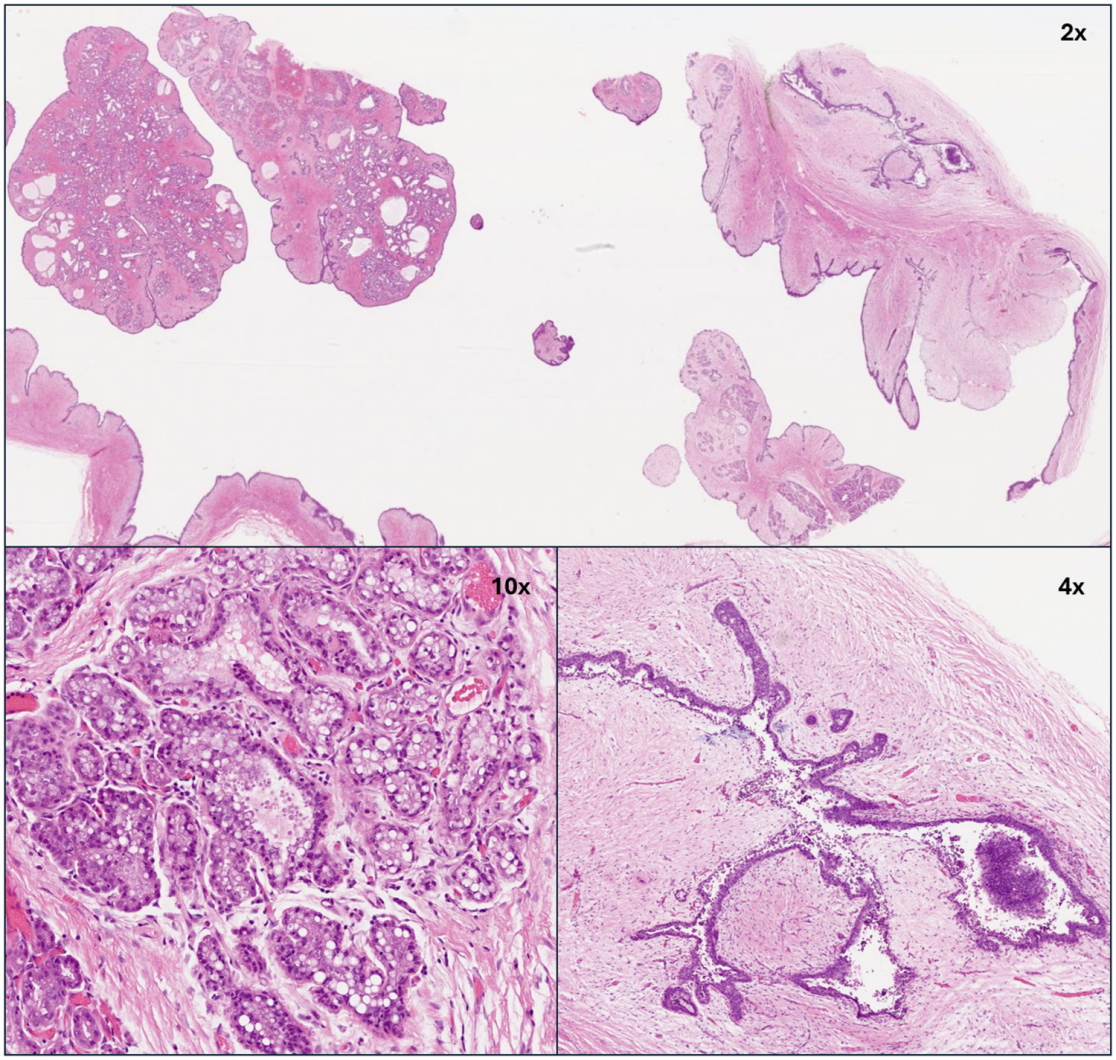
Lactating vulvar adenoma associated with a vulvar fibroadenoma. Low power (2×) magnification shows the entire lesion with the dual lactating adenoma (10× magnification) and fibroadenoma (4× magnification).

This lesion posed a diagnostic challenge. The differential diagnosis for the adenoma portion of the lesion included lactating adenoma tubular adenoma, lobular hyperplasia, carcinoma and apocrine adenoma. The presence of undeniable secretory changes as pictured in Figure 1 was not in favor of tubular adenoma. To argue against carcinoma, p63 (TP63) staining was done, which illustrated a myoepithelial layer. The differential diagnosis of the fibroepithelial portion of the lesion included fibroadenoma and phyllodes tumor. The absence of criteria for a phyllodes tumor (stromal atypia, high stromal cellularity, stromal overgrowth, etc.) was compatible with the diagnosis of fibroadenoma.

Positivity for GATA3, KRT7 with negative KRT20, as well as focal ER (ESR1) and BRST-2 (PIP) aligned with the breast differentiation of the lesion whereas CDX2, PSA (KLK3) and PAX8 were used to argue against other sites of origin. The presence of myoepithelial cells was shown by their staining with p63 and SMMHC, enforcing the benign nature of the lesion (Table 1).

**Table 1.** Immunohistochemistry Findings.

	Lactating adenoma	Fibroadenoma
ER	Focal (luminal cells)	Negative
CD10 (MME)	Positive (myoepithelial cells)	Positive (myoepithelial and stromal cells)
KRT7	Positive (epithelial cells)	Positive (epithelial cells)
KRT20	Negative	Negative
GATA3	Positive (epithelial cells)	Positive (epithelial cells)
p63	Positive (myoepithelial cells)	Positive (myoepithelial cells)
BRST-2	Focal (epithelial cells)	Focal (epithelial cells)
CDX2/PSA/PAX8	Negative	Negative

## Discussion

**Figure 2.**
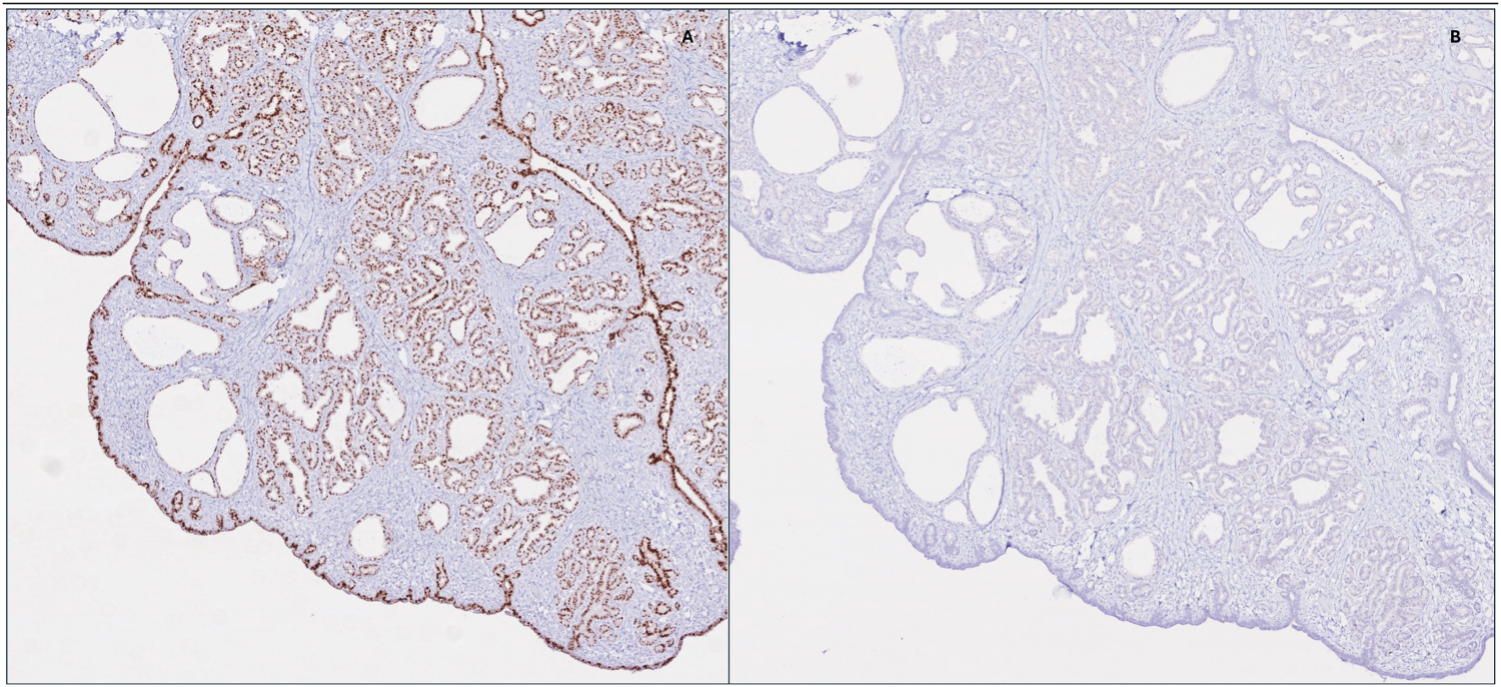
Immunohistochemistry was performed to confirm breast tissue differentiation (2A: GATA3, original magnification 4×) and to argue against endometrial origin (2B: PAX8, original magnification 4×).

**Figure 3.**
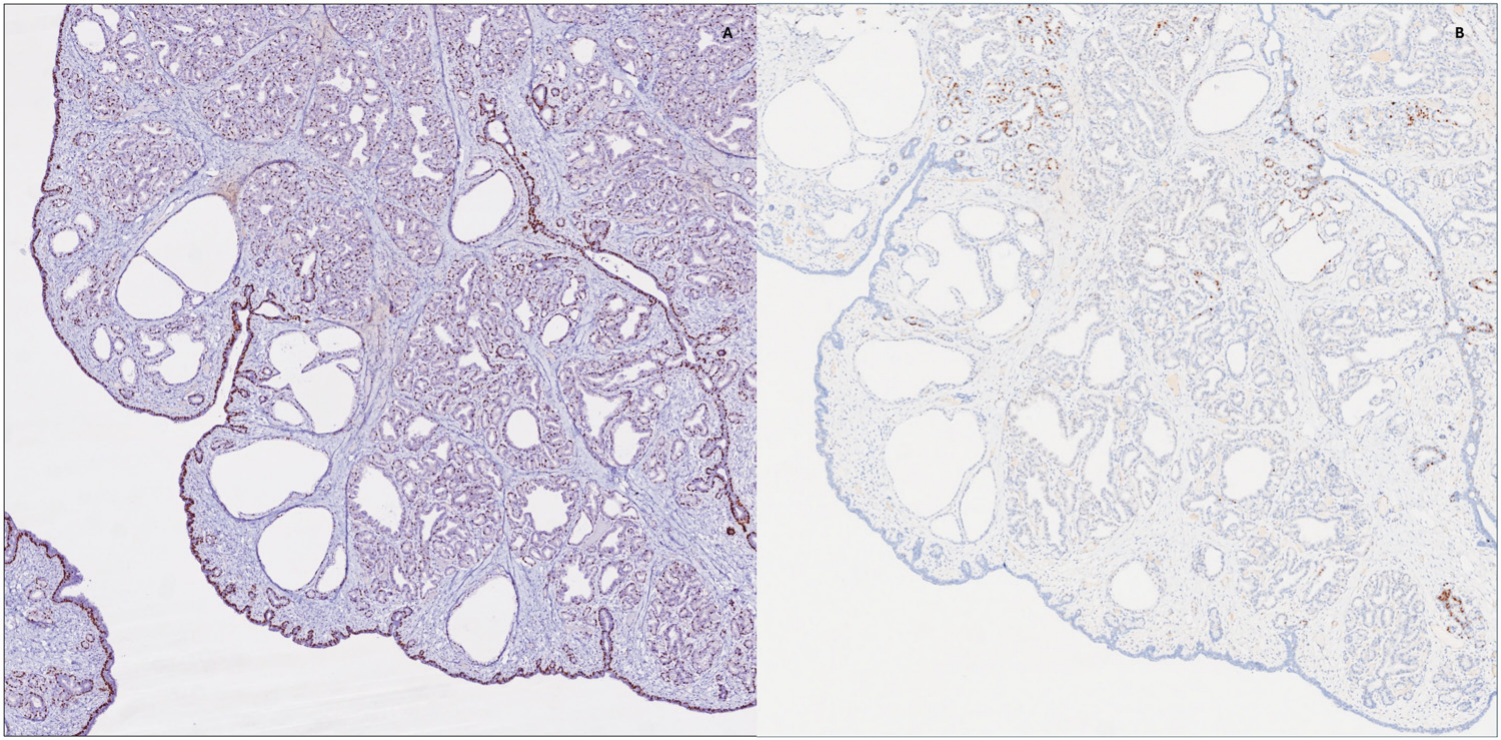
Immunohistochemistry was performed to highlight myoepithelial cells and enforce the benign nature of the lesion (3A: p63, original magnification 4×). ER expression was only focally positive (3B: ER, original magnification 4×).

The main pitfall for this lesion arises from its rare nature and glandular morphology, which can be interpreted as adenocarcinoma especially on frozen section examination. Appreciation of the lack of cytonuclear atypia and identification of remaining ectopic tissue as well as consideration of the age of the patient and postpartum status all assist in making the correct diagnosis. Myoepithelial stains should be performed to argue against invasive breast carcinoma. Lastly, when examining a fibroepithelial lesion arising on ectopic breast tissue, features of phyllodes tumor should be ruled out before arriving to a fibroadenoma diagnosis (Figures 2 and 3).

Ectopic breast tissue has been extensively documented in the literature,^5^ however, lesions arising on such tissues remain to be additionally studied. This report expands on the currently limited literature on vulvar lactating adenoma associated with vulvar fibroadenoma.^3,6–8^ The practicing pathologist should be wary of the possibility of mammary-like lesions occurring in the vulva and their possible variations to avoid misdiagnosis.
